# Trigeminal Trophic Syndrome as a Complication of Herpes Zoster Ophthalmicus

**DOI:** 10.7759/cureus.30382

**Published:** 2022-10-17

**Authors:** Daniela Tehfi, Alejandro Barrera-Godínez, Judith Dominguez-Cherit, Michelle Gatica-Torres

**Affiliations:** 1 Clinical Sciences, Tec Salud, Monterrey Institute of Technology and Higher Education (Instituto Tecnológico y de Estudios Superiores de Monterrey), Mexico City, MEX; 2 Dermatology, Salvador Zubirán National Institute of Health Sciences and Nutrition (Instituto Nacional de Ciencias Médicas y Nutrición Salvador Zubirán), Mexico City, MEX

**Keywords:** herpes zoster ophthalmicus, neuropathy, neuralgia, trigeminal trophic syndrome, herpes zoster

## Abstract

Trigeminal trophic syndrome (TTS) is an unusual complication that occurs secondary to trigeminal nerve injury. The insult to the nerve can lead to anesthesia, hypoesthesia, and paresthesias producing sensations such as burning or itching. The combination of both leads to repeated self-inflicted skin trauma in an attempt to alleviate these sensations, eventually leading to ulceration of the skin. We report a case of a 71-year-old male patient with a scalp ulcer who had an episode of herpes zoster ophthalmicus four months prior to presentation.

## Introduction

Trigeminal trophic syndrome (TTS) is a rare condition that commonly occurs as a consequence of trigeminal nerve injury [1]. The most common causes of TTS are iatrogenic such as therapeutic trigeminal nerve ablation. However, there are many other potential causes such as ischemic medullary or pontine stroke or herpes zoster infection. The damage to the trigeminal nerve causes facial dysesthesia and hypoaesthesia or even anesthesia that induce a self-mutilating behavior to alleviate sensations such as itching, burning, tingling, and crawling, which contribute to cutaneous injury and ulcer development [1-5].

## Case presentation

**Figure 1:**
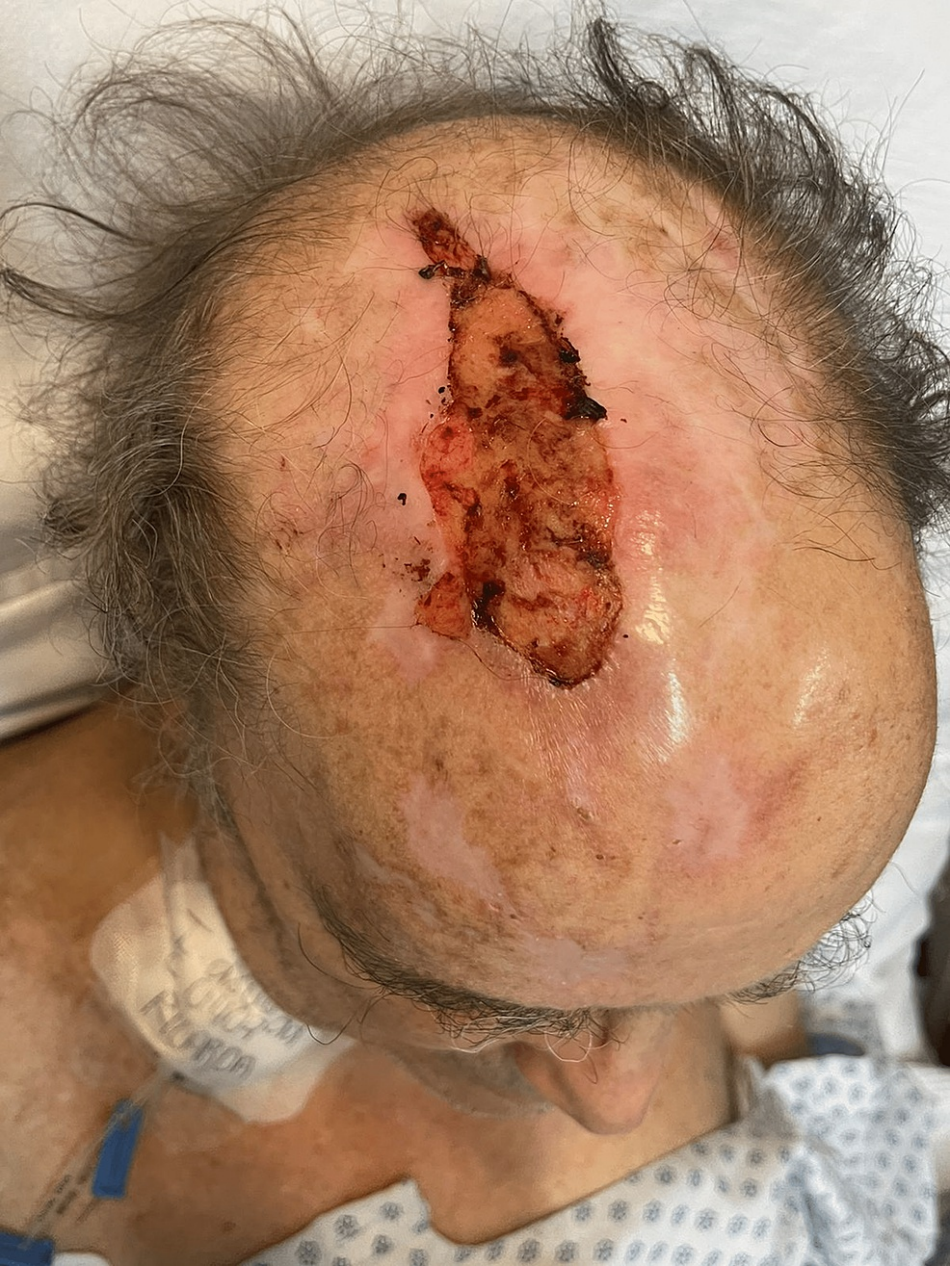
A superficial ulcer covered with granulation tissue with well-defined borders located along the right frontoparietal scalp. Hypopigmented scars secondary to skin lesions of herpes zoster are also present on the area of the right ophthalmic branch of the trigeminal nerve.

A 71-year-old male patient with type 2 diabetes admitted for orchiepididymitis, epididymal abscesses, and scrotal infection was evaluated by the dermatology team for a scalp ulcer that started developing a month prior. On physical examination, an 8 x 3.5 cm oval ulcer affecting the right frontoparietal scalp, with irregular and well-defined borders was noted. The ulcer’s surface was covered with granulation tissue and some crust. Perilesional skin had mild erythema, sun damage, and atrophy, as well as multiple hypopigmented atrophic scars, which appeared after a herpes zoster episode affecting the ophthalmic branch of the trigeminal nerve four months earlier (Figure 1). The area was frankly hypoesthetic and the patient had been suffering from paresthesias. The patient was diagnosed with postherpetic neuralgia weeks ago, which had been treated with 300 mg of pregabalin daily with poor response. 

Based on the location of the lesion, history of herpes zoster and postherpetic neuralgia, and the triad of ulceration, paresthesia, and hypoaesthesia, the diagnosis of TTS was established. The wound was covered with a hydrocolloid patch and changed every 48 hours with complete healing of the ulcer after a period of eight weeks.

## Discussion

TTS is a rare cause of skin ulceration secondary to trigeminal nerve pathway damage. The precise incidence is not known. It apparently lies somewhere between 150 and 200 cases according to two recent reviews of the syndrome [4,6].

The characteristic clinical triad of TTS is facial skin ulceration, paresthesia, and hypoesthesia or anesthesia [1-7]. There are multiple causes for TTS and the most common are iatrogenic such as therapeutic trigeminal nerve ablation. There are other potential causes including, but not limited to, ischemic medullary or pontine stroke, craniofacial surgery, acoustic neuroma, post-infectious encephalitis, astrocytoma, meningioma, surgical or local trauma, and amyloid deposits in the trigeminal nerve and herpes zoster infection, as is the case for this patient [1-7]. Most ulcers affect the infraorbital nerve distribution. The area most frequently affected is the nasal ala, which is supplied by one of the terminal cutaneous branches of the infraorbital nerve [2,3]. The period between trigeminal nerve insult and ulceration ranges from weeks to decades [1,4,5]. It’s more common in women, and the mean age across different publications tends to fall within the sixth and seventh decades [1,3-6, 8].

The mechanism for ulcer development is unclear. The contribution of neurotrophic factors and an altered sympathetic activity is thought to be involved and a pivotal role of self-mutilation is generally accepted [8]. One retrospective review and case series report mention that not all patients reported a self-mutilating behavior, suggesting other mechanisms influence ulcer development other than physical injury to the skin [3]. However, it is more likely they were either unaware of the self-manipulation or there was an external factor causing repetitive trauma unbeknownst to the patient.

There is no established treatment for TTS. The treatment is based on three pillars: behavioral modifications, wound care, and pharmacological treatment [1]. Some pharmacological therapies aiming at reducing paraesthesias have been attempted with variable success and limited evidence including carbamazepine, pregabalin, gabapentin, amitriptyline, pimozide, chlorpromazine, benzodiazepines, and topical tacrolimus [1-2]. Transcutaneous electric nerve stimulation (TENS), which aims to improve blood supply and healing of the area, has also been reported as a possible treatment [2,8]. Local application of neurotrophic factors and the transplantation of in vitro cultured epidermal cells have been successful in isolated cases [8]. The surgical management of TTS remains controversial. The use of contralateral, sensate flaps and a staged surgical approach appears to be effective [6].

## Conclusions

TTS is an unusual complication that occurs secondary to trigeminal nerve injury. Herpes zoster is one of many causes of trigeminal nerve injury that can lead to TTS. It’s important to keep TTS in mind as a complication of herpes zoster infection because, even though it’s not one of its most common causes, one in three people will have herpes zoster in a lifetime and post-herpetic neuralgia is one of its most common complications.
